# Lower gastrointestinal bleeding in a male with jejunal Dieulafoy's lesion after successful surgical resection: A case report and literature review

**DOI:** 10.1097/MD.0000000000029474

**Published:** 2022-06-24

**Authors:** Liang-Ying Chen, Yu-Han Hong, Shao-Ciao Luo, Jing-Tong Fu, Sz-Iuan Shiu

**Affiliations:** a Department of Internal Medicine, Taichung Veterans General Hospital, Taichung, Taiwan; b Institute of Medicine, Chung Shan Medical University, Taichung, Taiwan; c Department of Surgery, Taichung Veterans General Hospital, Taichung, Taiwan; d Department of Pathology, Taichung Veterans General Hospital, Taichung, Taiwan; e Division of Gastroenterology and Hepatology, Department of Internal Medicine, Taichung Veterans General Hospital, Taichung, Taiwan; f Department of Critical Care Medicine, Taichung Veterans General Hospital, Taichung, Taiwan; g Taiwan Association for the Study of Small Intestinal Diseases, New Taipei City, Taiwan; h Evidence-based Practice and Policymaking Committee, Taichung Veterans General Hospital, Taichung, Taiwan.

**Keywords:** case report: Dieulafoy's lesion, gastrointestinal bleeding, small bowel bleeding

## Abstract

**Introduction:**

Dieulafoy's lesion (DL) presented with small bowel bleeding constitutes a group of rare and potentially life-threatening prognosis. Several case series have described this condition, yet it remains unclear as to what is the optimal treatment and predicted outcome for patients who have been diagnosed.

**Patient concerns:**

We present a 21-year-old male experiencing bloody stool for 1 day.

**Diagnosis:**

Computed tomography of the abdomen exhibited active contrast extravasations and segmental wall thickening in the jejunum, and enteroscopy showed one 15-millimeter sized subepithelial tumor at the proximal jejunum.

**Interventions:**

Due to unstable vital signs he received an emergent transcatheter arterial embolization, and surgeon performed a laparoscopic surgical resection thereafter under the impression of potential malignancy. The pathologist confirmed jejunal DL with organizing thrombus.

**Outcomes:**

He was discharged on the 8th day of hospitalization without recurrent bleeding.

**Conclusion:**

A systematic literature review of 98 published cases taken from PubMed dating back to 1978 was undertaken, and the patients with DL and small bowel bleeding involved mainly the jejunum, followed by the duodenum and ileum. Meanwhile, DL-related duodenal bleeding was diagnosed mostly by an enteroscopy, as well as endoscopic interventions. Jejunal and ileal bleeding due to DL was surveyed through endoscopy and surgery, while surgical resection remained the choice for bleeding cessation. Only anticoagulant use (OR = 18.16; *P* = .08) was associated with a higher risk of overall mortality, although it was non-significant in univariate analysis. We emphasize that individualized treatment as well as prompt measurement should be implemented accordingly.

## 1 Introduction

Dieulafoy's lesion (DL) is a rare and potentially life-threatening disease,^[1]^ accounting for 3.5% of overall gastrointestinal bleeding and an overall mortality rate ranging from 23 to 79%.^[2,3]^ DL constitutes 15% and 1% of overall Dieulafoy's bleeding in the duodenum and jejunum, respectively.^[2]^ Prior to 1990, diagnosis and prompt intervention were challenging for patients experiencing small bowel bleeding due to DL, with most patients being treated surgically. Over the past 30 years, endoscopic management has become the mainstream therapy for DL-related small bowel bleeding, as manifested in one meta-analysis highlighting the efficacy between endoscopic band ligation and endoscopic hemoclip placement.^[4]^ However, the epidemiology, optimal treatment, and prognosis in these patients remain elusive.

In this report, we present a case of lower gastrointestinal bleeding in a male with jejunal DL after prompt endoscopic management and successful surgical resection, who has provided informed consent for publication of the case. In addition, we also analyzed the epidemiology, treatment, and prognosis of small bowel bleeding due to DL from material taken in a systematic literature review.

## 2 Case report

**Figure 1:**
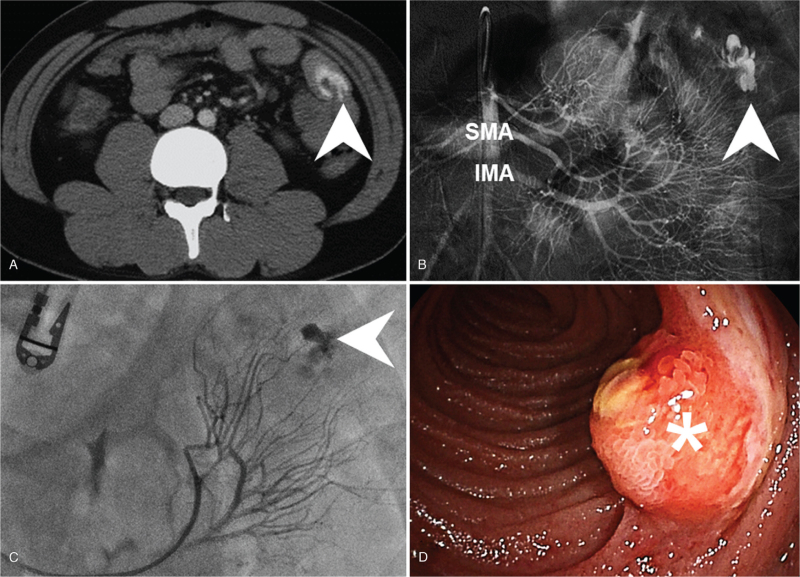
Active contrast extravasations and segmental wall thickening of jejunum (arrowhead) detected by computed tomography of abdomen with biphasic contrast enhancement (A). Emergent transcatheter arterial embolization performed immediately and after angiographic confirmation of active bleeding from branch of superior mesenteric artery (B) superselective embolization completed by gelform cubes injection (arrowhead) (C). A 15 millimeters subepithelial tumor at proximal jejunum (asterisk) with ulceration centrally and hyperemia peripherally observed via double balloon enteroscopy (D). SMA: superior mesenteric artery, IMA: inferior mesenteric artery.

**Figure 2:**
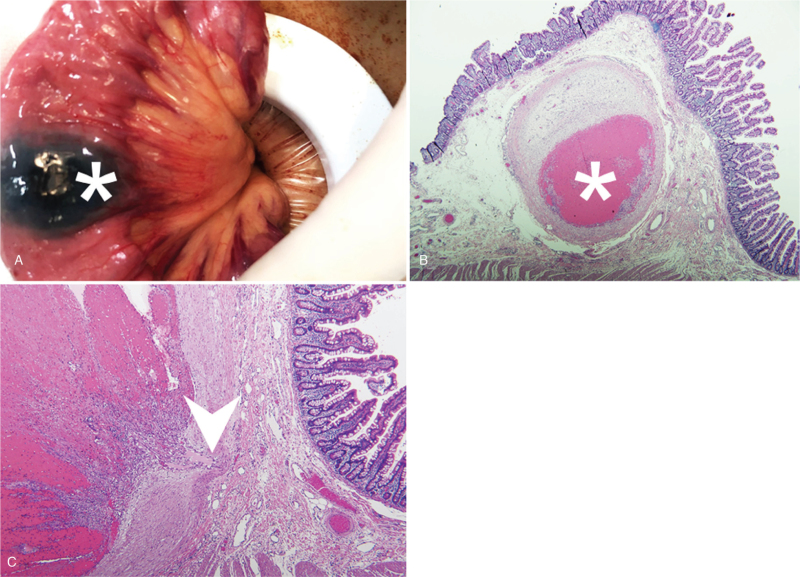
A laparoscopic surgical resection showed the location of subepithelial tumor with tattoo. (A). Jejunal Dieulafoy's lesion with organizing thrombus (asterisk) (B) and nearby nutritional vessel (arrowhead) (C) confirmed by pathology.

A 21-year-old male was presented to the emergency department with an acute-onset of bloody stool over the course of one day. The patient also reported simultaneous exertional dyspnea and dizziness. He recalled two episodes of painless bloody stool in the past 3 weeks, and colonoscopy had been performed twice without any specific bleeding being found. He denied any medical history related to his condition or any recent pharmacological use. Upon physical examination at admission his temperature was 35.9°C, heart rate 107 beats/minute, respiratory rate 18 breaths/minute and blood pressure 90/51 mmHg. An abdominal examination revealed a hyperactive bowel sound during auscultation and a soft sensation while performing palpation without any muscle guarding or rebounding pain. The remainder of the physical examination was unremarkable with the exception of a pale conjunctiva pallor. A laboratory examination revealed a total leukocyte count of 17,560 cells per μL, Hemoglobin concentration of 7.3 g/dL, and platelet count of 429,000 per μL. The patient's serum creatinine was 101.7 μmol/L, Internal Normalized Ratio (INR) 1.09, and activated Partial Thromboplastin Time (aPTT) 20.5 seconds on initial presentation. A chest X-ray examination was normal, while an electrocardiogram reported sinus tachycardia. Computed tomography of the abdomen with biphasic contrast enhancement exhibited active contrast extravasations and segmental wall thickening in the jejunum (Fig. 1A). Owing to the associated symptoms and unstable vital signs an emergent Transcatheter Arterial Embolization (TAE) was performed immediately. Subsequently, after angiographic confirmation of active bleeding from a branch of the superior mesenteric artery, superselective embolization was completed through gelfoam cubes injection (Fig. 1B, C). After admission to the general ward in stable condition we arranged for a painless double-balloon enteroscopy for the purpose of obtaining a differential diagnosis on the 5th day of hospitalization. This procedure revealed a 15-millimeter sized subepithelial tumor at the proximal jejunum, with ulceration centrally and hyperemia peripherally (Fig. 1D). We consulted surgeons regarding laparoscopic surgical resection being performed the following day under the impression of potential malignancy and a high risk of recurrent bleeding (Fig. 2A). The patient was then discharged on the 8th day of hospitalization. A pathologist then confirmed jejunal DL with organizing thrombus (Fig. 2B, C), and no recurrent bleeding was noted thereafter.

## 3 Discussion

Small bowel bleeding due to DL is uncommon but remains an important issue in clinical management, as indicated in a total of 98 cases being reported in the available literature [File S1, Supplemental Digital Content]. We extracted individual data via PubMed using keywords and synonyms, including “Dieulafoy's lesion”, “Duodenum”,” Jejunum” and “Ileum”. Among a total of 304 literature found, 98 cases having been published since 1978 and collected from the accessible literature without any language limitations contained a complete narration of diagnosis and treatment course. The location of DL was on the duodenum in 36.7% of the cases, the jejunum in 48%, and the ileum in 15.3% of the patients in our analysis. The mean patient age at admission was approximately 50.5 years, with a male predominance (62.5%). Melena and hematochezia accounted for the majority of initial presentations, with the overall mortality rate being 1.0%, and only one case of DL-related jejunal bleeding recorded. Within the DL-related duodenal bleeding patients, 67.6% were presented with melena, whereas 86.1% were diagnosed through an endoscopy [File S2, Supplemental Digital Content]. The interventions were approached using endoscopic hemostasis therapy. Additionally, the mean age 45.5 years in patients with DL-related jejunal bleeding was slightly younger and their condition mostly manifesting with melena and hematochezia. Patient diagnosis and management were both conducted through surgery, followed by endoscopy and angiography with therapeutic embolization based on our analysis. The mortality rate within DL-related jejunal bleeding was 2.1%. Patients with DL-related ileal bleeding were younger than 50 years, with a female predominance (57.14%) and initially expressed hematochezia (71.42%). Amongst these patients, endoscopy and surgery were the most common methods for diagnosis and treatment. A univariate analysis was performed based upon individual patient data, with the pooled analysis used to form a cohort of 98 patients in order to clarify any possible predictive factors associated with overall morality (Table 1). There was no significant difference in the univariate analysis except for anticoagulant use, which was shown to be associated with a higher risk of overall mortality with marginal significance (OR = 18.16, 95% CI, 0.69–479.41; *P* = .08).

**Table 1:** Univariate analysis of the clinical parameters related to mortality in patients with intestinal Dieulafoy's lesion.

	Total	Univariate		
		Odds ratio	95%CI	*P*-value
Age	91			
<65 years of age	56	Reference	Reference	
≥65 years of age	35	4.91	0.19–124.02	0.33
Gender	90			
Women	35	Reference	Reference	
Men	55	0.21	0.01–5.23	0.34
Comorbidity	54			
None	10	Reference	Reference	
Coagulopathy	4	9.00	0.29–275.58	0.21
Pharmacology	87			
None	57	Reference	Reference	
Anti-platelets	12	–	–	–
Anticoagulant	10	18.16	0.69–479.41	0.08
Organ dysfunction	23			
None	2	Reference	Reference	
Yes	21	0.37	0.01–11.63	0.57
Location	92			
Duodenum	34	Reference	Reference	
Jejunum	43	2.44	0.10–61.69	0.59
Ileum	15	–	–	–
Treatment				
Endoscopy	46	Reference	Reference	
Radiology	3	4.33	0.15–127.31	0.40
Surgery	42	0.36	0.01–9.00	0.53

Small bowel bleeding due to DL usually reveals concealed and intermittent gastrointestinal bleeding with an absence of surrounding mucosal ulceration.^[5]^ Endoscopic criteria for the diagnosis of DL consists of the presence of spurting or pulsatile bleeding from a small mucosal defect, protruding vessels with or without active bleeding, or fresh bleeding clots with a small adherence to mucosal defects surrounded by the normal appearance of mucosa.^[5,6]^ The epidemiology of DL-related small bowel bleeding varies greatly within the available literature consisting of case reports and case series.^[2,5,6]^ Additionally, gastrointestinal bleeding caused by DL is usually encountered in the stomach (72%), followed by the duodenum (15%), esophagus (8%), colon (2%), and rectum (2%). Recently, two literature reviews have discussed DL-related small bowel bleeding in the duodenum and jejunum with 38 and 136 cases being reported, respectively.^[6,7]^ Inayat et al^[6]^ reported that the mean age of patients with DL-related duodenal bleeding was 56 years with initial symptoms of melena and hematemesis, which is similar to our analysis. However, some patients were associated with an intermittent and massive hemorrhage leading to hemodynamic instability, where 2 out of 38 patients died due to cardiac and pulmonary complications. Regarding the therapeutic management, 23.7% received surgery while 76.3% were treated with endoscopic hemostasis. Within the patients treated conservatively, 62.1% received combinations of endoscopic therapies, including local injection, thermal or argon plasma coagulation, and mechanical instruments such as banding and hemoclipping. Duodenal DL is relatively difficult to treat in terms of its diagnosis and management due to several factors, including differentiation between DL and peptic ulcer disease, missed diagnosis due to adherent blood clots, periampullary diverticulum or sharp duodenal angulations, as well as obstacles to overcome beyond the second portion of the duodenum.

Malik et al^[7]^ demonstrated that the mean age of patients with jejunal DL was 55 years with a male predominance, and having initial symptoms of melena, obscure-overt gastrointestinal bleeding, and hemodynamic compromise, which was slightly different to our patients who were at younger age (50.5 years) during initial diagnosis. Endoscopic hemostasis was achieved in 64% of patients, while 34% of physicians preferred a combined therapy treatment involving two or more endoscopic modalities. The rebleeding rate was 13.4% and overall mortality rate 4.4%. Jejunal DL continues to be a diagnostic and therapeutic challenge depending upon the time interval between initial presentation and diagnostic modality, navigation of the jejunum, intermittent and indolent course of hemorrhage, and skillset of the doctor involved in device-assisted endoscopy, as well as other factors. Although the standard diagnostic and therapeutic modalities remain elusive, performing device-assisted enteroscopy with hemostasis may be the first priority rather than radiographic or angiographic modalities in patients without hemodynamic instability.^[8,9]^ The rebleeding rate after endoscopic treatment seemed higher in jejunal DL when compared to duodenal DL, as mentioned by Yilmaz et al^[2]^.

Although the baseline characteristics of our patients were not standardized in our review, we do offer descriptive information of the patients in DL-related small bowel bleeding according to different locations. To our knowledge, this is the first comprehensive literature review regarding small bowel bleeding related to DL discussing epidemiology, optimal treatment, and prognosis. Our review emphasizes the need for both prompt evaluation and individualized treatment in order to achieve improved clinical outcomes in patients with DL-associated small bowel bleeding via use of multidisciplinary teamwork.

## Acknowledgment

We would like to express our appreciation to the assistance provided by the Evidence-based Practice and Policymaking Committee of Taichung Veterans General Hospital when preparing this report.

## Author contributions

S-SI, C-LY, and H-YH conceptualized the case report and literature review. C-LY, H-YH, S-SI, L-SC, and F-JT contributed to data curation, formal analysis, investigation, methodology, and project administration. C-LY, H-YH, and S-SI wrote the original draft, performed the writing, review, and editing of the manuscript, and approved the final draft of the article.

**Conceptualization:** Sz-Iuan Shiu, Liang-Yin Chen, Yu-Han Hong

**Data curation:** Liang-Yin Chen, Sz-Iuan Shiu, Yu-Han Hong, Jing-Tong Fu, Shao-Ciao Luo

**Formal analysis:** Sz-Iuan Shiu, Liang-Yin Chen

**Investigation:** Sz-Iuan Shiu, Liang-Yin Chen

**Methodology:** Sz-Iuan Shiu, Liang-Yin Chen

**Project administration:** Sz-Iuan Shiu, Liang-Yin Chen

**Writing – original draft:** Liang-Yin Chen, Sz-Iuan Shiu

**Writing – review & editing:** Sz-Iuan Shiu, Liang-Yin Chen

## Supplementary Material

Supplemental Digital Content

## Supplementary Material

Supplemental Digital Content
