# Supplementary material for: Lower gastrointestinal bleeding in a male with jejunal Dieulafoy's lesion after successful surgical resection: A case report and literature review
Source: Medicine (Baltimore). 2022 Jun 24;101(25):e29474. doi: 10.1097/MD.0000000000029474 (PMC9276124; doi:10.1097/MD.0000000000029474)
Supplement: Supplemental Digital Content [file medi-101-e29474-s001.doc]

**Supplementary Table 1.** The demographic data of patients with intestinal Dieulafoy’s lesion in literature review

| **Author** | **Year** | **Age** | **Gender** | **Initial presentation (Melena=0, Hematochezia=1, Hematemesis=2)** | **Comorbidity (Coagulopathy=0, Thrombocytopenia=1, Others=2)** | **Pharmacology**  **(Anti-platelets=0, Anticoagulant=1, NOAC=2, Others=3, None=4)** | **Location (Duodenum=0, Jejunum=1, Ileum=2, Combined=3)** | **Diagnostic tools**  **(Confirmed; attempted)** | **Times from presentation to diagnosis (days)** | **Hospitalization (days)** | **Treatment (Cured)** | **Mortality** |
| --- | --- | --- | --- | --- | --- | --- | --- | --- | --- | --- | --- | --- |
| Yamauchi N, et al. | 2020 | 45 | M | 1 | 2 | 3 (Steroid) | 1 | Laparoscopy with intraoperative enteroscopy; CTA | NA | NA | Surgical resection | No |
| Kieswetter L, et al. | 2019 | 9 | F | NA | 2 | 4 | 1 | Exploratory laparotomy with intraoperative endoscopy; CTA, SBCE, DBE | NA | 60 | Surgical resection | No |
| Kawabata H, et al. | 2019 | 81 | M | 0 | 2 | 0+3 | 1 | UGI, LGI; Contrast CT | NA | NA | Clips | No |
| Scimeca D, et al. | 2019 | 69 | M | NA | NA | 4 | 0 | UGI | NA | NA | Clips | NA |
| Beatrice P, et al. | 2019 | 73 | M | 0 | 2 | No | 0 | Exploratory laparotomy; UGI | NA | NA | Vessel ligation | No |
| Saada M, et al. | 2019 | 27 | M | 1 | NA | 4 | 1 | Scintigraphy; UGI, LGI, Angiography | 4 | 9 | Surgical resection | No |
| Zhao J, et al. | 2019 | 41 | M | 1 | No | No | 1 | SBE; UGI, LGI | 3 | NA | Surgical resection | No |
| Jung C, et al. | 2019 | 69 | NA | 0 | No | No | 1 | SBE; UGI, LGI | NA | NA | Clips | No |
| Becq A, et al. | 2018 | 32 | F | 1 | No | 3 | 2 | Exploratory laparotomy with intraoperative enteroscopy; UGI, LGI, CTA | NA | NA | Surgical resection | No |
| Mai D, et al. | 2018 | 83 | F | 0 | 2 | 0 | 2 | LGI; UGI | NA | NA | Clips | No |
| Komissarov IA, et al. | 2018 | 1 | M | 2 | NA | 4 | 0 | UGI; LGI | 9 | 23 | Endovascular embolization | No |
| Satyavada S, et al. | 2018 | 55 | M | 0+2 | 2 | 4 | 0 | EUS; UGI, LGI, SBCE, push enteroscopy, RBC scan | NA | NA | Epinephrine injection, Clips | No |
| Soria Alcívar M, et al. | 2018 | 60 | M | 0+1 | NA | 4 | 0 | UGI | NA | NA | Clips | NA |
| de Benito Sanz M, et al. | 2018 | 82 | M | 0 | NA | 1 | 0 | UGI | NA | NA | Epinephrine injection, Clips | No |
| Relea Pérez L, et al. | 2018 | 91 | M | 2 | NA | 1 | 0 | UGI | NA | NA | Epinephrine, Aethoxysklerol, Clips | No |
| Seo KI, et al. | 2017 | 25 | M | 1 | No | No | 1 | Exploratory laparotomy, SBCE; Angiography, UGI, LGI | NA | NA | Surgical resection | No |
| Holleran G, et al. | 2016 | 75 | F | 0 | 2 | 1 | 0 | DBE; UGI, LGI, SBCE | NA | NA | Ileohepatic artery bypass | No |
| Holleran G, et al. | 2016 | 67 | F | 0 | 0 | 1 | 1 | DBE; UGI, LGI, CT mesenteric angiogram, SBCE, surgery | 730 | NA | APC, Clips, IM somatostatin analogue | Yes |
| Holleran G, et al. | 2016 | 74 | F | 0 | 2 | 1 | 1 | SBCE; UGI, LGI | 540 | NA | APC | No |
| Holleran G, et al. | 2016 | 76 | F | 0 | 0 | 1 | 1 | DBE; SBCE, mesenteric angiogram, surgery | 360 | NA | Surgical resection | No |
| Sathyamurthy A, et al. | 2016 | 63 | M | 0 | 2 | 1 | 0 | UGI; LGI, RBC scan | NA | NA | Clips | No |
| Aoyama T, et al. | 2016 | 79 | M | 0 | 2 | 0 | 1 | DBE; UGI, LGI, CTA, SBCE | 30 | NA | APC, Clips | No |
| Chen IS, et al. | 2016 | 54 | M | 1 | 2 | 4 | 2 | Balloon-assisted enteroscopy; UGI, LGI, CTA | NA | NA | Surgical resection | No |
| Lipka S, et al. | 2015 | 69 | F | 0 | 2 | 0 | 0 | SBE; UGI, LGI, SBCE | NA | 6 | Bipolar EC, Clips | No |
| Lipka S, et al. | 2015 | 60 | F | 0 | 2 | No | 1 | SBE; UGI, LGI, SBCE | NA | 7 | Bipolar EC, Clips | No |
| Lipka S, et al. | 2015 | 60 | F | 0 | 2 | 0 | 1 | SBE; UGI, LGI, SBCE | NA | 3 | APC | No |
| Lipka S, et al. | 2015 | 73 | F | 0 | NA | 4 | 1 | SBE; UGI, LGI, SBCE | NA | 2 | Bipolar EC | No |
| Lipka S, et al. | 2015 | 63 | M | 0 | 2 | 0 | 1 | SBE; UGI, LGI, SBCE | NA | 6 | Bipolar EC, Epinephrine injection, Clips | No |
| Lipka S, et al. | 2015 | 80 | M | 0 | 2 | 0 | 1 | SBE; UGI, LGI, SBCE | NA | 4 | Bipolar, Epinephrine injection | No |
| Lipka S, et al. | 2015 | 81 | M | 0 | 2 | 0 | 1 | SBE; UGI, LGI, SBCE | NA | 7 | Bipolar EC | No |
| Lipka S, et al. | 2015 | 86 | M | 0 | 2 | 0+1 | 1 | SBE; UGI, LGI, SBCE, TAE | NA | 27 | Bipolar EC, APC, Clips | No |
| Kozan R, et al. | 2014 | 21 | F | 1 | 2 | 4 | 1 | Exploratory laparotomy with intraoperative endoscopy; Angiography | 3 | 16 | Surgical resection | No |
| Gauci J, et al. | 2014 | 74 | M | 0 | 2 | 1 | 0 | UGI; RBC scan, LGI | NA | NA | Surgical oversuture | No |
| Çolak B, et al. | 2014 | 21 | M | 2 | No | No | 1 | Angiography, intraoperative endoscopy; UGI | NA | NA | Surgical resection | No |
| Alomari AI, et al. | 2013 | 14 | F | 2 | NA | 4 | 0 | Angiography; UGI, push endoscopy, SBCE | NA | NA | Embolization | No |
| Nadal E, et al. | 2013 | 41 | F | 0 | NA | 4 | 0 | UGI | NA | NA | Cyanoacrylate injection | No |
| Gomerčić Palčić M, Ljubičić N. | 2013 | 61 | F | 0 | NA | No | 0 | UGI | NA | 4 | Loop ligation | No |
| Han MS, et al. | 2013 | 54 | M | 1 | 2 | No | 1 | Pathological finding; UGI, LGI | NA | 6 | Surgical resection | No |
| Choi YC, et al. | 2012 | 79 | F | 1 | 2 | 0 | 2 | SBE; UGI, LGI, CT | NA | NA | Clips | No |
| Rao S, et al. | 2012 | 3 | M | 1 | NA | 4 | 0 | Exploratory laparotomy; UGI, LGI | NA | NA | Surgical resection | No |
| Mohamad A, et al. | 2012 | 67 | M | 0+2 | 2 | 4 | 0 | UGI | NA | NA | Epinephrine injection, Clips | No |
| Choi YC, et al. | 2012 | 47 | M | 1 | NA | No | 2 | SBE; CT, angiography, UGI, LGI | NA | NA | Clips | No |
| Shibutani S, et al. | 2011 | 14 | F | 1 | NA | 4 | 2 | Pathologic finding; UGI, CT, angiography | NA | NA | Surgical resection | No |
| Madono K, et al. | 2011 | 78 | F | 2 | 2 | 4 | 2 | LGI; CT | NA | NA | Clips | No |
| Chung CS, et al. | 2011 | NA | NA | 0 | 2 | 4 | 1 | SBE; UGI, angiography | 3 | NA | APC, Clips | No |
| Rana SS, et al. | 2010 | 52 | F | 0 | No | No | 0 | ERCP; UGI, LGI, SBCE, push enteroscopy | NA | NA | Epinephrine injection | No |
| Coumaros D, Tsesmeli N. | 2010 | 86 | F | 0 | NA | 0 | 0 | UGI | NA | NA | Monopolar EC | No |
| Ezzat RF, et al. | 2010 | 7 | F | 0 | No | No | 2 | LGI | NA | NA | Surgical resection | No |
| Ujiki MB, et al. | 2010 | 48 | M | 2 | NA | 4 | 0 | Laparotomy; UGI, LGI, angiography, RBC scan | NA | NA | Surgical resection | No |
| Folgado Alberto S, et al. | 2010 | 64 | M | 0 | 2 | 0 | 0 | Exploratory laparotomy; UGI | NA | NA | Epinephrine, Aethoxysklerol, APC | No |
| Sadio A, et al. | 2010 | 75 | M | 1 | 0 | 4 | 0 | UGI | 4 | 11 | Cyanoacrylate injection | No |
| Saji N, et al. | 2010 | 72 | M | 0 | 2 | 4 | 1 | UGI; LGI, CT, angiography | NA | NA | TAE | No |
| A. Macrì, et al. | 2009 | 68 | F | 0 | NA | 4 | 0 | UGI; Angiography | NA | NA | APC | No |
| Moreira-Pinto J, et al. | 2009 | 14 | F | 2 | No | 4 | 1 | Exploratory laparotomy; UGI, LGI, angiography | 1 | NA | Surgical resection | No |
| Marangoni G, et al. | 2009 | 15 | F | 0+2 | No | No | 1 | Exploratory laparotomy with intraoperative enteroscopy; CTA, UGI | NA | NA | Surgical resection | No |
| Yang CW, et al. | 2009 | 54 | M | 1 | No | No | 1 | DBE; UGI, LGI, CT, angiography | NA | NA | Clips | No |
| Lim YJ, et al. | 2008 | 36 | M | 0 | NA | 0 | 1 | UGI; CT angiography, SBCE | 21 | NA | Surgical resection | No |
| Sai Prasad TR, et al. | 2007 | 13 | M | 0 | NA | 4 | 1 | Laparoscopic-assisted transumbilical resection; UGI, scintigraphy, SBCE | NA | NA | Surgical resection | No |
| Min En Nga, et al. | 2007 | 39 | M | 1 | NA | No | 1 | CTA, laparotomy; UGI, LGI | 7 | NA | Surgical resection | No |
| Tsai CL, et al. | 2007 | 28 | M | 1 | NA | 4 | 2 | Angiography; UGI, LGI | NA | NA | Surgical resection | No |
| Ko KH, et al. | 2005 | 79 | F | 0+1 | NA | 4 | 0 | UGI | 3 | 17 | Clips | No |
| Marchese M, et al. | 2005 | 65 | M | 0 | 0 (VWD)+1 | 4 | 0 | UGI; LGI, SBCE | NA | NA | Epinephrine, APC | No |
| Anagnostopoulos G, et al. | 2005 | 74 | M | 0 | NA | 4 | 0 | Endoscopy | NA | NA | Clips | No |
| Kim JK, et al. | 2005 | 35 | M | NA | NA | 4 | 1 | Exploratory laparotomy; UGI, LGI, mesenteric angiography, enteroclysis | NA | NA | Surgical resection | NA |
| Mino A, et al. | 2004 | 31 | F | 0 | NA | 4 | 1 | Laparoscopic resection; UGI, LGI, mesenteric angiography, CT | NA | NA | Surgical resection | No |
| Morowitz MJ, et al. | 2004 | 4 | M | 1 | NA | 4 | 2 | Pathological findings, enteroclysis; Merckel’s scan, RBC scan, LGI, UGI | NA | NA | Surgical resection | No |
| Iglesias SS, et al. | 2004 | 68 | M | 1 | NA | 4 | 2 | LGI | NA | NA | Clips | No |
| Cleary M, et al. | 2004 | 46 | M | NA | NA | 4 | 2 (Merckle's diverticulum) | Pathological findings; UGI, LGI | NA | NA | Surgical resection | No |
| Ibrarullah M, et al. | 2003 | 25 | F | 0 | NA | 4 | 0 | UGI | NA | NA | Epinephrine | No |
| Lee BI, et al. | 2003 | 70 | F | 2 | NA | 4 | 0 | UGI | 1 | 12 | Clips | No |
| Ibrarullah M, et al. | 2003 | 31 | M | 2 | 2 | 4 | 0 | UGI | NA | NA | Epinephrine | No |
| Owaki T, et al. | 2002 | 12 | F | 1 | NA | 4 | 1 | Exploratory laparotomy; UGI, LGI, mesenteric angiography | NA | NA | Surgical resection | No |
| Ueno N, et al. | 2002 | 37 | M | 1 | NA | 4 | 1 | Exploratory laparotomy with intraoperative enteroscopy; UGI, LGI, mesenteric angiography | 13 | NA | Surgical resection | No |
| Fox A, et al. | 2001 | 47 | F | 0 | 2 | 4 | 2 | Pathological findings; UGI, LGI | NA | NA | Surgical resection | No |
| Blecker D, et al. | 2001 | 69 | F | 2 | 2 | 3 | 2 | Pathological findings; Bleeding scan, enteroscopy, angiography | NA | NA | Surgical resection | No |
| Blecker D, et al. | 2001 | 18 | M | 1 | NA | 4 | 1 | Exploratory laparotomy with intraoperative enteroscopy; UGI, LGI, scintigraphy, mesenteric angiography | 5 | 12 | Surgical resection | No |
| Fallows GA, et al. | 2000 | 19 | F | 0+1 | NA | 3 (NSAID) | 1 | Exploratory laparotomy; UGI, LGI, CT, barium contrast small bowel, mesenteric angiography | 9 | 13 | Surgical resection | No |
| Overgaard H, et al. | 2000 | 54 | F | 1 | NA | 4 | 1 | Exploratory laparotomy | NA | NA | Surgical resection | NA |
| Gadenstätter M, et al. | 1998 | 32 | M | NA | NA | NA | 0 | Enteroscopy | NA | NA | Aethoxysklerol injection | No |
| Inaguma D, et al. | 1998 | 54 | M | 0 | 2 | NA | 0 | Enteroscopy | NA | NA | Steroid | No |
| Deutsch G, et al. | 1998 | 27 | M | 1 | NA | 4 | 1 | NA | NA | NA | NA | NA |
| Gadenstatter M, et al. | 1998 | 45 | M | 0+1 | 2 | 4 | 1 | Exploratory laparotomy; UGI | NA | NA | Surgical resection | No |
| Gadenstatter M, et al. | 1998 | 54 | M | 0+1 | 2 | 4 | 1 | Exploratory laparotomy; UGI | NA | NA | Surgical resection | No |
| Geschwind JF, et al. | 1998 | 73 | M | NA | NA | 4 | 1 | Exploratory laparotomy; Scintigraphy | NA | NA | Surgical resection | NA |
| Lee, KS, et al. | 1997 | 20 | F | 1 | NA | 4 | 1 | Exploratory laparotomy; UGI, LGI, scintigraphy, angiography | 1 | 9 | Surgical resection | No |
| Sueoka N, et al. | 1997 | 28 | M | 1 | NA | NA | 0 | Enteroscopy | NA | NA | Clips | No |
| Sueoka N, et al. | 1997 | 50 | M | 1 | NA | NA | 0 | Enteroscopy | NA | NA | Clips | No |
| Nishida K, et al. | 1997 | 79 | M | 0 | NA | 4 | 0 | Enteroscopy | 7 | NA | Hypertonic saline epinephrine | No |
| Murray KF, et al. | 1996 | 12 | M | 2 | 2 | 0 | 1 | UGI | NA | NA | Epinephrine, Elastic band | No |
| Goins WA, et al. | 1995 | 36 | M | 1 | NA | 3 (Drug abuse) | 1 | Exploratory laparotomy (2nd); LGI, scintigraphy, exploratory laparotomy with intraoperative enteroscopy (1st) | 15 | NA | Surgical resection | No |
| Raijman I, et al. | 1995 | 72 | M | NA | 2 | NA | 2 | LGI | NA | NA | Thermocoagulation | No |
| Choudari CP, et al. | 1993 | 55 | M | 0 | 2 | 1 | 0 | UGI | NA | NA | Epinephrine | No |
| Goldenberg SP, et al. | 1990 | 68 | F | 0 | 2 | 1 | 0 | UGI | NA | NA | Epinephrine, Cauterization | No |
| Golding MI, et al. | 1990 | 81 | F | 1 | NA | 4 | 0 | UGI; Nuclear medicine | NA | NA | Cauterization | No |
| Vetto JT, et al. | 1989 | 58 | M | 0+1 | 2 | 3 (Drug abuse) | 1 | Exploratory laparotomy; UGI, LGI | 1 | 10 | Surgical resection | No |
| Vetto JT, et al. | 1989 | 72 | M | 0 | 2 | 4 | 1 | Exploratory laparotomy; UGI, barium enema, angiography | NA | NA | Surgical resection | No |
| Matuchansky C, et al. | 1978 | 24 | M | 0 | 2 | 4 | 1 | Exploratory laparotomy; UGI, LGI | 15 | NA | Surgical resection | No |
| Matuchansky C, et al. | 1978 | 31 | M | 0+1 | NA | 4 | 1 | Exploratory laparotomy (2nd); UGI, exploratory laparotomy (1st) | 11 | NA | Surgical resection | No |
| Mean |  | 50.5 | **62.5%M**  **37.5%F** | **53.1% Melena**  **35.7% Hematochezia 14.3% Hematemesis**  **7.1% NA** | **10.2% No**  **4.1% Coagulopathy 1% Thrombocytopenia 40.8% Others**  **44.9% NA** | **64.3% No**  **14.3% Anti-platelet 11.2% Anticoagulant 0% NOAC**  **7.1% Others** | **36.7% Duodenum 48% Jejunum 15.3% Ileum** |  | 74.8 [1-730] | 12.6  [2-60] | **51% Endoscopic hemostasis**  **44.9% Surgery**  **3.1% TAE**  **1% NA** | 1.0% |
