# Supplementary material for: Lower gastrointestinal bleeding in a male with jejunal Dieulafoy's lesion after successful surgical resection: A case report and literature review
Source: Medicine (Baltimore). 2022 Jun 24;101(25):e29474. doi: 10.1097/MD.0000000000029474 (PMC9276124; doi:10.1097/MD.0000000000029474)
Supplement: Supplemental Digital Content [file medi-101-e29474-s002.doc]

**Supplementary Table 2.** Characteristics of patient with intestinal Dieulafoy’s lesion in literature review

| Patient characteristics | Duodenum  (n = 36) | Jejunum  (n = 47) | Ileum  (n = 15) |
| --- | --- | --- | --- |
| Age (years) | 57.8 | 45.5 | 48.5 |
| Male (%) | 63.9 | 66.7 | 46.7 |
| Initial presentation (%) | | | |
| Melena | 67.6 | 59.1 | 23.1 |
| Hematochezia | 20.6 | 45.5 | 61.5 |
| Hematemesis | 23.5 | 9.1 | 15.4 |
| Time from presentation to diagnosis (days) | 1-9 | 1-730 | NA |
| Diagnostic tools (%) | | | |
| Endoscopy | 86.1 | 30.4 | 53.3 |
| Capsule endoscopy | 0 | 8.7 | 0 |
| Surgical intervention | 11.1 | 54.3 | 40 |
| Angiography | 2.8 | 4.3 | 6.7 |
| Scintigraphy | 0 | 2.3 | 0 |
| Interventions (%) | | | |
| Endoscopic hemostasis | 77.8 | 32.6 | 40 |
| Transarterial embolization | 5.6 | 2.2 | 0 |
| Surgical resection | 13.9 | 65.2 | 60 |
| Others | 2.7 | 0 | 0 |
| Hospitalization (days) | 4-23 | 2-60 | NA |
| Hospital Mortality (%) | 0 | 2.1 | 0 |

NA: not available
